# Fluorescence-Based Monitoring of *In Vivo* Neural Activity Using a Circuit-Tracing Pseudorabies Virus

**DOI:** 10.1371/journal.pone.0006923

**Published:** 2009-09-09

**Authors:** Andrea E. Granstedt, Moriah L. Szpara, Bernd Kuhn, Samuel S. -H. Wang, Lynn W. Enquist

**Affiliations:** Department of Molecular Biology, Neuroscience Institute, Princeton University, Princeton, New Jersey, United States of America; Cedars-Sinai Medical Center and University of California Los Angeles, United States of America

## Abstract

The study of coordinated activity in neuronal circuits has been challenging without a method to simultaneously report activity and connectivity. Here we present the first use of pseudorabies virus (PRV), which spreads through synaptically connected neurons, to express a fluorescent calcium indicator protein and monitor neuronal activity in a living animal. Fluorescence signals were proportional to action potential number and could reliably detect single action potentials *in vitro*. With two-photon imaging *in vivo*, we observed both spontaneous and stimulated activity in neurons of infected murine peripheral autonomic submandibular ganglia (SMG). We optically recorded the SMG response in the salivary circuit to direct electrical stimulation of the presynaptic axons and to physiologically relevant sensory stimulation of the oral cavity. During a time window of 48 hours after inoculation, few spontaneous transients occurred. By 72 hours, we identified more frequent and prolonged spontaneous calcium transients, suggestive of neuronal or tissue responses to infection that influence calcium signaling. Our work establishes *in vivo* investigation of physiological neuronal circuit activity and subsequent effects of infection with single cell resolution.

## Introduction

Recording the activity of multiple neurons in intact circuits remains challenging for *in vivo* study because current tools cannot simultaneously report activity and connectivity. Electrophysiological recordings and fluorescent calcium indicators can inform about activity but not large-scale circuit connectivity. Genetic tools offer complementary ways of distinguishing and targeting cell types [1], but a combination of functional imaging and circuit tracing is still missing. Conversely, commonly used chemical tracers [2] and neurotropic viruses [3] can identify neuroanatomical circuits but do not document activity. An ideal tool should simultaneously report activity and connectivity with sensitivity and reliability for long periods of time *in vivo*.

Transient increases in calcium [Ca^2+^] levels are a useful correlate of neuronal activity. Calcium transients can be measured by injected dyes or by genetically encoded fluorescent [Ca^2+^] indicator proteins (FCIPs). G-CaMP2 is an FCIP composed of a circularly permuted green fluorescent protein (GFP) linked to calmodulin (CaM) and the CaM target sequence M13 [4]. In the presence of [Ca^2+^], CaM binds M13, resulting in increased fluorescence [5], [6]. The fluorescence intensity increases as a function of [Ca^2+^] concentration. G-CaMP2 is a calcium sensor that is stable at physiological pH and mammalian temperature [7]. Several publications have demonstrated the use of G-CaMP2 in mammals *in vivo* [8], [9].

Though FCIPs can reliably monitor neural activity, they need a vehicle to cross synapses and thereby report connectivity. Neurotropic viruses have proven useful to this end [3]. In particular, pseudorabies virus (PRV) has been used extensively for elucidating neural circuits in the peripheral and central nervous system *in vivo* [10], [11]. One variant, PRV-Bartha, is an attenuated, retrograde tracer that travels along chains of synaptically connected neurons, and many PRV-Bartha derivatives with fluorescent labels have been widely implemented for circuit tracing [12]. A recent publication reported the construction of a gE-deleted PRV-Kaplan recombinant containing the ratiometric calcium indicator TN-L15, and demonstrated its use by imaging explanted retina [13].

In PRV's history of use as a neuroanatomical tracer *in vivo*, studies have traditionally been done by infecting living animals and subsequently studying fixed and labeled tissues. Advances in imaging technology, such as two-photon microscopy [14], now raise the possibility of using PRV to deliver fluorescent labels that can be imaged in living animals. The submandibular ganglia (SMG) present an opportunity to implement these methods: a minor surgery allows optical access to these peripheral ganglia in living mice [15]. This preparation has recently been used to study synaptic contacts and receptor dynamics over time *in vivo* [16]. The combination of a fluorescent calcium sensor, delivered virally to optically accessible SMG neurons, provides a first opportunity to image PRV live *in vivo* to explore both connectivity and activity.

By inserting the calcium sensor G-CaMP2 into the genome of the classical PRV-Bartha tracing strain and monitoring activity *in vivo*, we present the first use of PRV to express a fluorescent calcium indicator protein and report fluorescence-based neuronal activity in the living animal. We confirmed that this recombinant, PRV369, could reliably detect single action potentials *in vitro*. We then studied fluorescence-based activity *in vivo* in the SMG. We identified a window of 48 hours after inoculation in which PRV369 can be used to look at calcium signals in these ganglia. Infected neurons responded to external electrical and sensory stimuli. After 72 hours we detected changes in intracellular [Ca^2+^] concentrations and duration of [Ca^2+^] transients, indicating cell or tissue responses to infection. PRV369 can be used for *in vivo* investigation of physiological neuronal circuit activity and subsequent effects of infection with single cell resolution.

## Results

### PRV369 infected cells express G-CaMP2

**Figure 1:**
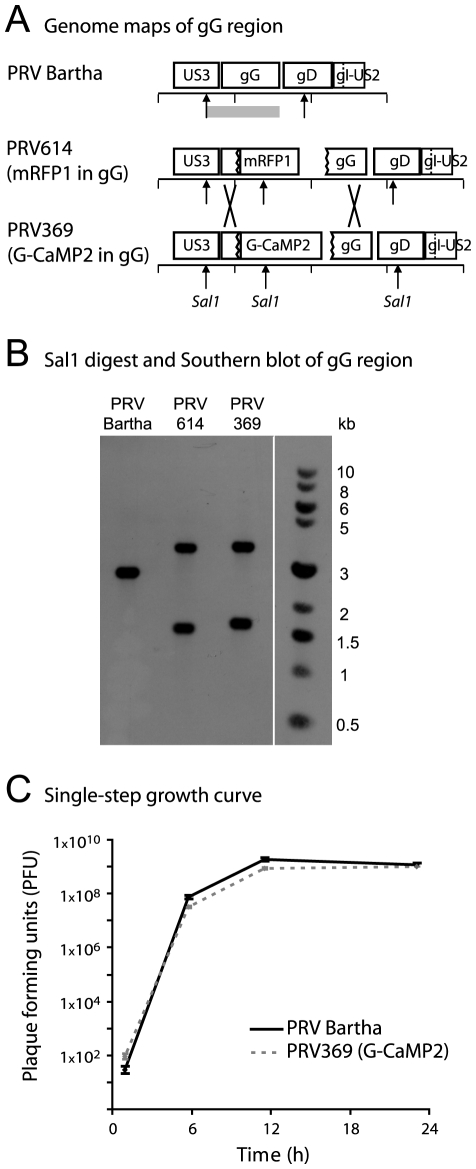
PRV369 stably encodes G-CaMP2 in the gG locus. (A) Map of the gG region of PRV, for the background strain PRV-Bartha, the mRFP1-containing derivative PRV614, and the G-CaMP2-containing derivative PRV369. The retrograde-limited spread phenotype of PRV Bartha stems from a deletion in this region, which results in the fused open reading frame of gI-Us2. The lines below the gene names are marked with 1 kb spacing. Cross marks indicate regions of homologous recombination used to generate PRV369. (B) Nucleocapsid DNA from strains PRV Bartha, PRV614, and PRV369 were digested with Sal1 and probed by Southern blot with a 1.9 kb fragment of Us3 and gG (gray box below Bartha genome in (A)). Fragments observed were the expected sizes, based on Sal1 cut sites denoted by arrows in (A): PRV Bartha fragment of 2.6 kb, PRV614 fragments of 1.5 and 3.4 kb, and PRV369 fragments of 1.6 and 3.5 kb. (C) Equivalent single-step growth kinetics of PRV Bartha and PRV369 in epithelial cells *in vitro*. PK-15 cells were infected at an MOI of 10, with input virus inactivated by a low pH citrate wash after 1 hour. Each time point was performed in triplicate and titered in duplicate. The average titer for each virus is plotted along with the standard error of the mean (SEM) for each time point and virus.

PRV369 was constructed by homologous recombination and replacement of mRFP1 in the glycoprotein G (gG) locus of PRV614, a PRV-Bartha-derived tracing strain (Figure 1A). Viral protein gG is not required for spread *in vivo* [17], and many of the commonly used PRV viral tracing strains employ insertions at this locus [18]. The G-CaMP2 open reading frame is transcribed from the cytomegalovirus immediate-early (CMV-IE) promoter (see [18] for more details). Correct genomic insertion of the G-CaMP2 cassette was confirmed by Southern blot analysis (Figure 1B). Western blots revealed no change in expression of upstream and downstream proteins (data not shown) in comparison to the parental PRV614 strain. PRV369 grows comparably to PRV-Bartha in cultured epithelial cells (Figure 1C).

### Fluorescence traces correlate with neuronal activity *in vitro*

**Figure 2:**
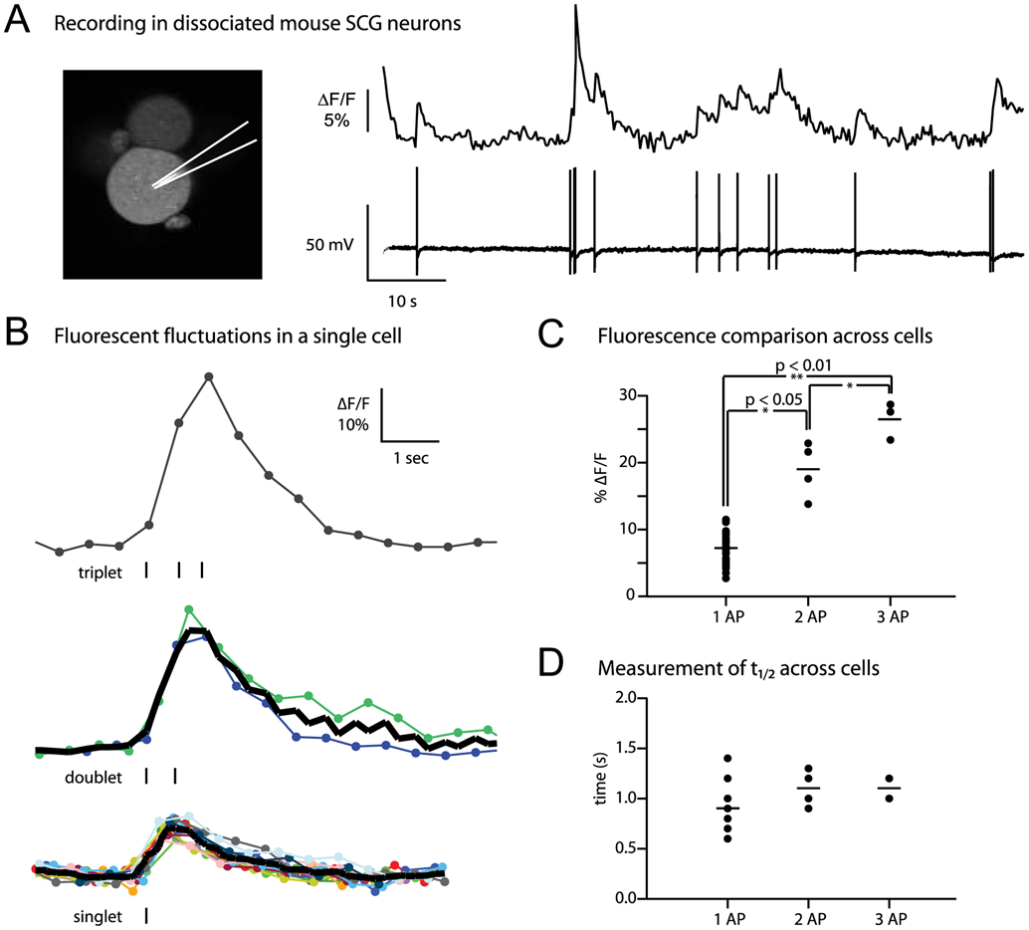
PRV369 sensitively and accurately detects graded neuronal activity in dissociated neurons *in vitro*. (A) Sample recording of an impaled neuron, with diagram indicating electrode position on an actual image of an SCG neuron (left). In response to every action potential (bottom trace), a calcium event was detected in the fluorescence trace (top trace). (B) Analysis for one neuron of the percent fluorescence change for single, double, or triple action potentials. Fluorescence increases proportionally to action potential number. Ticks indicate action potentials by electrophysiology. Bold line indicates average. (C) Quantitative comparison across multiple neurons. The amplitude of the fluorescence peak is significantly different between one, two, or three action potentials: the average ΔF/F was 7%, 19%, and 27%, respectively. (D) Comparison of fluorescence decay from peak. There is no statistical difference in decay between one, two, or three action potentials. The average t_1/2_ was 0.9, 1.1, and 1.1 seconds, respectively. AP, action potential; ΔF/F, relative fluorescence change.

To test whether changes in fluorescence correlated with firing of action potentials (APs), we infected dissociated mouse superior cervical ganglion (SCG) neurons and recorded action potentials with sharp electrodes while imaging corresponding fluorescence changes with two-photon microscopy. A typical infected SCG neuron is shown in Figure 2A. The electrical recording shows action potentials and the optical recording displays the corresponding [Ca^2+^] transients. We found that in all recordings, every action potential correlated with a change in fluorescence (representative trace in Figure 2A). This virally-delivered indicator reliably reported single action potentials *in vitro*.

We analyzed the amplitude of fluorescence fluctuations in response to action potential firing. Single action potentials were common, whereas bursts of two or more action potentials were scarce and irregular. Single, double, or triplet spikes could easily be separated in the [Ca^2+^] transients by the maximal relative fluorescence change (Figure 2B). In one cell we detected an average fluorescence change of 8.4±1.6% for single action potentials, 19.3±5.5% for spike doublets, and 32% for a triplet. We defined spikes as doublets and triplets if the interspike interval was less than 300 milliseconds (Figure 2B). Across four cells, we compared the average maximal amplitude of fluorescence change and found a statistically significant difference between fluorescence levels for one, two, and three action potentials (Figure 2C). We then measured the t_1/2_ from the peak, or the time it takes for the fluorescence to decay halfway from peak amplitude (Figure 2D). As expected, there was no statistically significant difference between one, two, and three action potentials (one vs. two, p = 0.1; two vs. three, p = 0.8), indicating that G-CaMP2 was not saturated with calcium. These *in vitro* characterizations ensured that this viral strain appropriately expressed and delivered G-CaMP2 in infected cells. Therefore we progressed to studying fluorescence-based activity under physiological conditions *in vivo*.

### Infection by PRV369 enables visualization of spontaneous activity *in vivo*

**Figure 3:**
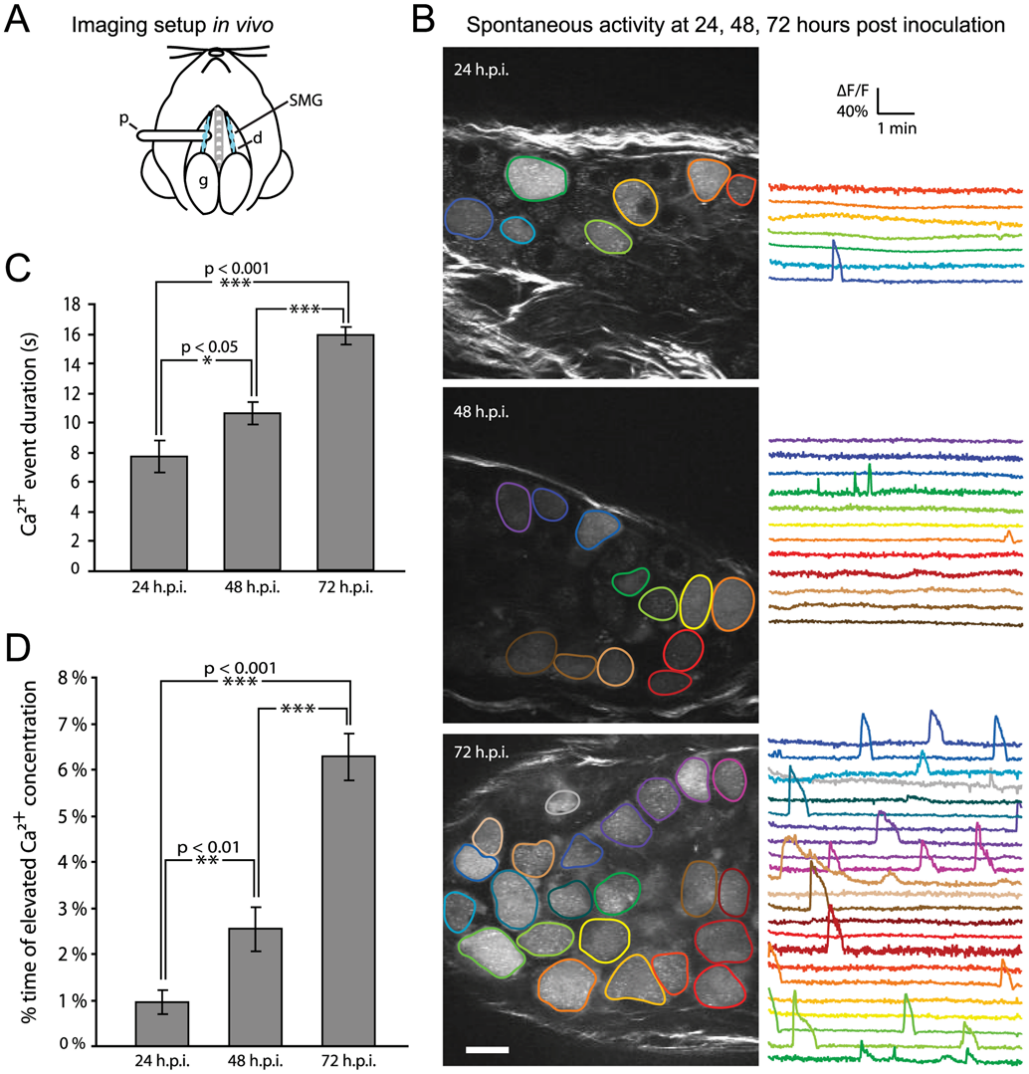
Infection with PRV369 reports spontaneous activity *in vivo*. (A) Diagram of setup for *in vivo* imaging. SMG (light blue) are located along the salivary duct (d) and send projections to the salivary glands (g) where the virus was injected. The platform (p) elevates the ganglia for imaging under two-photon microscopy. (B) Traces of spontaneous activity at 24, 48, and 72 hours post inoculation (h.p.i.). The number of infected cells increases with time, and there is also an increase in the number of calcium events. The average fluorescence change of these changes *in vivo* is 50%. Scale bar = 20 µm. (C) The average duration of a calcium event increases with the time after inoculation, with 72 h.p.i. exhibiting the most significant increase. The average duration is 7.6±1.1, 10.5±0.7, and 15.8±0.6 seconds for 24, 48, and 72 h.p.i., respectively. (D) The average percent of time that a cell is in a state of elevated intracellular calcium concentration also increases with time after inoculation. The average percent time was 1.0±0.3, 2.5±0.5, 6.3±0.5% for 24, 48, and 72 h.p.i., respectively. Labeled cells at 72 h.p.i. spend significantly more time in an elevated calcium state.

Using two-photon microscopy, we imaged the spontaneous activity in a known neuronal circuit *in vivo*. The submandibular ganglia (SMG) are peripheral parasympathetic ganglia that innervate the salivary glands and receive input from the superior salivatory nucleus in the brainstem; this circuit has already been defined in viral tracing experiments [19], [20]. We imaged the SMG in the anesthetized mouse at 24, 48, and 72 hours after PRV369 inoculation (Figure 3A-B and Supplemental Video S1). The ganglia are located along the salivary duct and are accessible for imaging *in vivo*. We perfused the area with warmed mammalian Ringer's solution and heated the animal to maintain physiological temperature. We used a miniature platform to raise ganglia away from surrounding tissues (Figure 3A), and only ganglia that were within a few millimeters of the gland were able to be lifted on the platform. Fluorescence changes related to spontaneous activity were easily detectable *in vivo* (Figure 3B). The number of infected neurons appeared to increase with time. To count the total number of labeled neurons, we pooled data from accessible ganglia in 3 animals at each time point. At 24 hours we counted a total of 65 labeled cells; at 48 hours, 133 cells; and at 72 hours, 280 cells. [Ca^2+^] signals were detectable at a high signal-to-noise ratio because of the high relative fluorescence change of G-CaMP2 upon [Ca^2+^]-binding [4]. These results show that infection by PRV369 was successful *in vivo* and allowed optical analysis of an infected neuronal circuit in a living animal.

### Time course of infection reveals changes in calcium activity

Infection by PRV-Bartha activates host defenses [21], [22], [23], which may influence the behavior of neurons. Therefore we sought to define an optimal window for using PRV369 to image native network activity in the SMG. We observed the rate of spontaneous calcium events at 24, 48, and 72 hours post inoculation (h.p.i.) in the salivary glands (Figure 3C). Analysis of the number of calcium events at each time point revealed a change in event frequency as time after infection increased: At 24 and 48 h.p.i., the majority of infected neurons generated events sparsely, but by 72 h.p.i., more cells were active and at higher rates (example in Figure 3B). In addition, we observed a statistically significant difference in the average duration of calcium events at 72 h.p.i. compared to 24 or 48 h.p.i. (Figure 3C). We further calculated the fraction of time that labeled cells spent at elevated intracellular calcium levels and observed a difference between 24 and 48 h.p.i., with a larger difference occurring at 72 h.p.i. (Figure 3D, see also Supplemental Figure S1). At 72 h.p.i. the cells displayed more complex patterns in the calcium events, indicative of unusually large bursts of activity (compare traces in Figure 3B). We conclude that in the SMG, PRV369 is most useful for measuring circuit activity within 48 hours after inoculation. Since G-CaMP2 fluorescence of infected SMG neurons appeared by 24 hours, there is a window of about 24 hours in which to observe and analyze neuronal activity with minimal effects of infection.

### Labeled SMG neurons reveal responses to electrical and sensory stimuli *in vivo*

**Figure 4:**
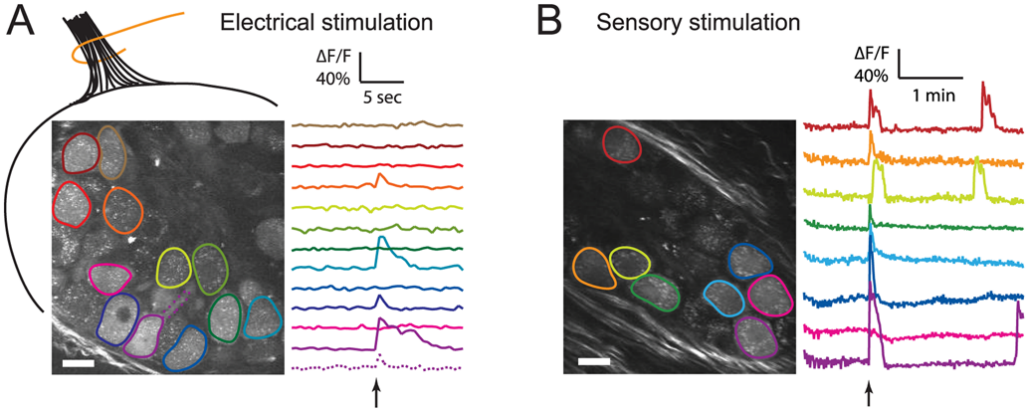
PRV369-infected SMG neurons respond to external electrical and sensory stimuli. (A) An electrical stimulus (arrow below traces) elicited a sharp and transient response in a subset of neurons. The diagram indicates where the tungsten electrode (orange) was placed on the innervating presynaptic axons from the brainstem (black bundle). Occasionally, the axon initial segment was visible (dotted purple trace) and triggered a shorter calcium event compared to the cell body of the same neuron (solid purple trace). (B) When hot distilled water (T = 40°C) was delivered to the oral cavity (arrow below traces) as a natural sensory stimulus for salivation, SMG neurons produced a strong and synchronous response. Scale bar = 20 µm.

We next used PRV369 infection to record stimulus-evoked (non-spontaneous) activity in the SMG *in vivo*. We electrically stimulated infected ganglia to evaluate the responsiveness of the infected neurons to external stimuli. We delivered an electrical pulse train via a wire electrode placed on the presynaptic axon bundle emanating from the brainstem to innervate the SMG. This stimulus triggered synchronous calcium transients in a subset of the neurons in the field of view (Figure 4A). Occasionally, an axon initial segment was clearly visible and also responded with a transient signal upon stimulation (Figure 4A, dotted purple trace). As expected, the calcium event triggered in the axon initial segment was of shorter duration, compared to the calcium event in the cell body of the same neuron (Figure 4A, solid purple trace). In general, ΔF/F values for electrically stimulated calcium transients were on average smaller and shorter-lasting than spontaneous transients, especially at later time points of infection. Moving the electrode to different position on the presynaptic axon bundle activated a different subset of neurons (data not shown). Antidromic stimulation yielded similar results, and neurons were responsive to electrical stimulation at 24, 48, and 72 hours after inoculation (data not shown).

We tested the sensory response of the SMG at 48 h.p.i. It has been reported that hot (T = 50°C) distilled water delivered to the oral cavity elicits a salivatory reflex [24]. We applied 40°C distilled water to the oral cavity and observed a sensory-evoked response in PRV369-labeled neurons. A subset of neurons exhibited a strong response (Figure 4B and Supplemental Video S2) and sensory-evoked events were of similar duration to spontaneous events. Similar groups of neurons fired during repetitions of the sensory stimulus (data not shown). In one animal, across three repeated sensory stimulations, 12 out of 15 fluorescent cells showed sensory responses; 2/3 of the responsive cells exhibited calcium transients 100% of the time (data not shown). Sensory responses were not seen when the oral cavity was stimulated with distilled water at room temperature (23°C) (data not shown), demonstrating that the responses were temperature-specific. Taken together, these results demonstrate the utility of PRV369 infection for analysis of electrically evoked responses and physiologically relevant sensory stimulation.

## Discussion

We have constructed a circuit-tracing PRV-Bartha derivative, PRV369, that expresses the fluorescent [Ca^2+^] indicator G-CaMP2. This virus provides the capability to reliably detect neuronal activity by fluorescent protein expression in intact circuits in living animals. We demonstrated its use *in vivo* for observing spontaneous and elicited neuronal activity in the peripheral nervous system, with a useful window of at least 24 hours from the first appearance of labeled neurons. PRV369 is isogenic with PRV152 (Bartha GFP) [17] and PRV614 (Bartha RFP) [18], two of the most frequently used PRV-based viral circuit tracers, facilitating the use of PRV369 in circuits already elucidated by these tracers. PRV369 can also be used in dual-infection experiments with these isogenic parental strains [10], [18], [25]. The prior use of PRV in delineating central nervous system (CNS) circuits (reviewed in [26] and [12]), and recent advances in live imaging with other genetic calcium indicators in the CNS (see for example [27] and [28]), suggest the potential usefulness of PRV369 for revealing activity in synaptically connected CNS neurons *in vivo*.

In contrast to PRV369, the recently published As1-PRV08 calcium sensor virus was constructed in a mutated PRV-Kaplan strain background, which the authors found to have higher infectivity and more cytotoxicity than several PRV-Bartha-derived strains tested in their study [13]. In addition, As1-PRV08 expresses the TN-L15 calcium sensor [29], a FRET-based [Ca^2+^] indicator. In matched *in vivo* comparisons [30], G-CaMP2 was found to have faster kinetics than TN-L15, including a 4-fold faster off-rate for calcium. In response to a strong stimulus, Boldogkoi et al. found an average 13% increase in the citrine∶CFP fluorescence ratio in ganglion cells infected by As1-PRV08 [13]. In SCG neurons infected with PRV369 *in vitro*, the average fluorescence change was 7% for a single action potential, and 27% for triplets. In the SMG *in vivo*, signals of up to 100% relative fluorescence change were observed.

SMG neurons could not be impaled for intracellular recording or bolus-loaded with calcium dyes *in vivo* without damaging the neurons. The SMG is surrounded by extracellular connective tissue, and each neuron is ensheathed by satellite cells [31]. Most studies have characterized SMG neuron activity with electrodes *ex vivo* (for example, [32]) or extracellular recording *in vivo* [33]. One published method for intracellular recording of SMG neurons under *in vivo* conditions necessitated a separate recording chamber and partial extraction of tissue [34]. PRV369 is thus noteworthy for allowing characterization of neuronal activity in a circuit that is otherwise difficult to access by previous recording methods. In the future, PRV369 should prove useful for studying local circuits in which multiple connected neurons are visible within the same field of view, allowing noninvasive and simultaneous characterization of network activity in multiple cells.

The endogenous activity of PRV369-labeled neurons can be monitored for at least 24 hours with limited effects due to infection. Most SMG neurons generated signals sparsely at 24 and 48 hours post inoculation into the salivary glands, consistent with previous published data that spontaneous firing is rarely observed from these cells [35]. However, the number of infected cells and firing frequency increased significantly by 72 hours. In addition, the average duration of a [Ca^2+^] event lasted significantly longer at late time points in infection. As suggested by cytological changes observed in other circuits, the later time point may correlate with the initiation of host defenses stimulated by infection [23]. The increased activity we observe at 72 hours post inoculation marks the first live, *in vivo* observation of these long-suspected effects of viral infection. One previous report monitored herpes simplex virus infection in transgenic report mice using titer-dependent bioluminescence techniques, but not at the level of single cells [36]. We envision that further experiments with PRV369 in infected circuits *in vivo* will allow exploration into the nature of the host response to infection. In summary, PRV369-labelling of connected neurons allows for reliable and sensitive detection of endogenous circuit activity early in infection, and will provide a long-sought means to simultaneously reveal both connectivity and activity at single cell resolution, in intact neural circuits *in vivo*.

## Methods

### Cells and virus construction

PRV 369 was constructed by homologous recombination between a plasmid containing a G-CaMP2 expression cassette and the gG locus of the PRV-Bartha genome. The plasmid pN1-G-CaMP2 (pN1-RSET-mG1.6#X-1) was a gift of Dr. Junichi Nakai [4], and contains the coding sequence of G-CaMP2 in place of EGFP in the pEGFP-N1 plasmid, under the control of a cytomegalovirus immediate-early (CMV-IE) promoter (Clontech; GenBank Accession # U55762). A 990 bp fragment of PRV gG was isolated by PstI and NdeI digestion of the previously described pBB04 plasmid [17]. This vector was cloned into the DraIII site of pN1-G-CaMP2, downstream of the simian virus 40 (SV40) polyadenylation sequences. This plasmid was linearized and co-transfected into swine epithelial (PK15) cells with DNA from PRV614, a PRV Bartha derivative with mRFP1 inserted into the gG locus [18]. Homologous recombination of the G-CaMP2 cassette into the gG locus of PRV614 occurred between the CMV-IE promoter and the flanking gG sequence, replacing the original 2.3 kb mRFP cassette at this locus with the 2.5 kb G-CaMP2 cassette. Resulting recombinants were plaque-purified on PK15 cells by selection of non-red plaques. Southern blot analysis using a 1.9 kb piece of gG (EcoRI – HindIII fragment) as a probe confirmed that the GCaMP2 cassette had recombined correctly into the PRV genome, producing the recombinant PRV369. Restriction fragment length polymorphism (RFLP) analysis using BamHI, KpnI, and PstI was used to compare PRV Bartha, the parental PRV614, and PRV369, confirming the correct integration of the G-CaMP2 cassette (data not shown). Western blot for the upstream gene Us3 and downstream gene gD revealed no changes in expression of these proteins relative to the parental PRV614 strain (data not shown). PRV614 and related tracing strains have a previously described decrease in Us3 expression relative to PRV-Bartha [37].

### Dissociated neuronal cultures of superior cervical ganglia (SCG)

SCG neurons were used for *in vitro* characterization of PRV369 for ease of maintenance in dissociated culture. Methods for harvesting and culturing SCGs have been described in detail elsewhere [38]. Briefly, SCG ganglia were harvested from mouse embryos at embryonic day 15, dissociated, and allowed to settle on tissue culture grade plates coated with 0.5 mg/ml poly-ornithine and 10 µg/ml laminin. Neurons were cultured in Neurobasal™ media containing, 1X Penicillin-Streptomycin-Glutamine solution, 1X B-27 supplement, and 50 ng/ml nerve growth factor (all from Invitrogen), and allowed to mature for 10 days before using in experiments.

### Electrophysiology

Experiments *in vitro* were performed at room temperature. We recorded spontaneous activity from 16 to 18 hours after infection. The effect of PRV infection on dissociated superior cervical ganglia neurons has been recently characterized (McCarthy, Tank, Enquist, submitted). For intracellular recordings, quartz micropipettes were pulled (P-2000, Sutter Instrument, Novato, CA) to a resistance of 40–60 MΩ and filled with 10 µM fluorescein in 3 M KCl. Recordings were acquired at 5 kHz (Axopatch 200B, Axon Instruments).

### Infection of mouse submandibular ganglia (SMG)

All animal procedures were performed in accordance with the guidelines of the National Institutes of Health and were approved by local authorities (Princeton University Institutional Animal Care and Use Committee). The model for mouse SMG infection was previously described in detail [39]. Briefly, mice aged 3–5 months were anesthetized with a mixture of ketamine (100 mg/kg) and xylazine (10 mg/kg) by intraperitoneal administration. A midline incision was made along the neck, exposing the salivary glands. Using a Hamilton syringe, concentrated PRV inoculum (10^10^ pfu/ml) was injected into both submandibular glands (5 µl/side). The incision was sutured and the mice were given a dose of buprenorphrine (100 µg/kg concentration) against post-surgical pain. The mice were allowed to recover for various times post inoculation.

### Calcium imaging in vivo

The protocol for imaging infected SMG in living mice was adapted from Purves and Lichtman [15], [40]. Mice were anesthetized in an isofluorane chamber and then transferred in supine position to a custom-built stage with heating pad, rectal temperature probe (FHC Inc.), and nose cone for isoflurane inhalation. A ventral midline incision was made along the neck and the skin was pulled back with retractors. The SMG were exposed and lifted on a small custom-made metallic platform, controlled by a micromanipulator. The area was perfused with warmed mammalian Ringer's solution containing (in mM): 154 NaCl, 5.6 KCl, 2.4 NaHCO_3_, 2 Tris buffer pH 8.0, and 2.2 CaCl_2_, adjusted to pH 7.4 with HCl. The animal's temperature was maintained at 35°C.

### Electrical and sensory stimulation

We used a tungsten electrode (1 MΩ resistance) to electrically stimulate pre- and post-synaptic axons by 10 pulses of 60 V, delivered every 0.1 ms at 100 Hz (ISO-Flex triggered by Master-8, A.M.P.I.). For the sensory stimulation, we injected aliquots (300 µl) of hot water (40°C at outlet) at a pressure of 8 psi to the mouth by air-pressure from a picospritzer (Pressure System IIe, Toohey Company) while simultaneously imaging the SMG. The tubing was heated electrically.

### Two-photon microscopy

Two-photon fluorescence imaging was performed on a custom-built microscope with a tunable Mira 900 Titanium∶sapphire laser (Coherent) at 910 nm exciation wavelength and a GaAsP photomultiplier tube (H7422P-40, Hamamatsu). The microscope was controlled by CfNT software (M. Müller, Max Planck Institute for Medical Research, Heidelberg, Germany). We used a 40x, 0.8 N.A. water-immersion objective (HCX APO, Leica) to record movies with 256×256 or 128×128 pixel at 2 ms/line scan rate.

### Image analysis

The two-photon raw data was first opened in ImageJ (W. S. Rasband, ImageJ, National Institutes of Health, Bethesda, Maryland, http://rsb.info.nih.gov/ij/). We defined an infected neuron as 30% brighter than background fluorescence from an area outside of the ganglia in the same field of view. We established this threshold because uninfected cells have punctate autofluorescence intensities that range from 10–20% above background. A region of interest was drawn around each infected cell, and the mean intensity over time was saved and further analyzed using Igor Pro 6 software (WaveMetrics, Inc., http://www.wavemetrics.com). For ΔF/F measurements in plots, the background fluorescence was calculated by fitting a curve. Peaks were identified using a custom-written program in Python (Python Software Foundation). The analysis program used algorithms based on a previous publication [41]. The program identified a baseline and recognized peaks as points that were 0.75 intensity units above the baseline and 10% above baseline noise ratio.

## Supporting Information

Figure S1 Probability distribution that the occurrence of a calcium event will last a certain length of time (in seconds) for 24, 48, or 72 hours post inoculation (h.p.i.). At 72 h.p.i., the occurrence of long-lasting calcium events is more frequent than earlier time points, and only at this time point do calcium events last longer than 50 seconds.
(0.01 MB PDF)

Video S1 Spontaneous activity of submandibular ganglia (SMG) neurons, expressing G-CaMP2 delivered by PRV369. Movies were acquired at 256×256 pixels and 2 ms/line for 800 frames, and played back at 40 frames per second (20 times sped up). The movies were recorded at 24, 48, or 72 hours post inoculation, in order of appearance.
(7.96 MB MOV)

Video S2 Stimulus-evoked activity revealed in labeled SMG neurons, expressing G-CaMP2 delivered by PRV369. Movies were acquired at 256×256 pixels and 2 ms/line, for 50 frames in electrical stimulus and 400 frames in sensory stimulus, and played back at 20 and 40 frames per second (10 and 20 times sped up), respectively. Both movies were recorded at 48 hours post inoculation. The white rectangle indicates the time of stimulus. To reduce the amount of movement, the sensory-evoked stimulus movie was stabilized with ImageJ plugin: (http://www.cs.cmu.edu/~kangli/code/Image_Stabilizer.html).
(2.98 MB MOV)
