# Supplementary figures and images for: Fluorescence-Based Monitoring of In Vivo Neural Activity Using a Circuit-Tracing Pseudorabies Virus
Source: PLoS One. 2009 Sep 9;4(9):e6923. doi: 10.1371/journal.pone.0006923 (PMC2735035; doi:10.1371/journal.pone.0006923)

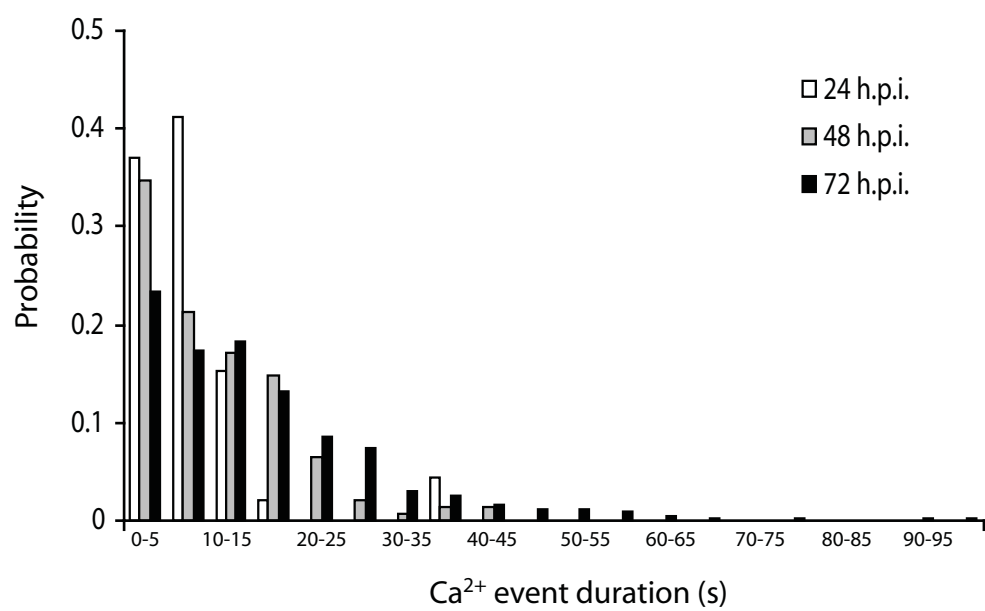

Supplement: Figure S1 — Probability distribution that the occurrence of a calcium event will last a certain length of time (in seconds) for 24, 48, or 72 hours post inoculation (h.p.i.). At 72 h.p.i., the occurrence of long-lasting calcium events is more frequent than earlier time points, and only at this time point do calcium events last longer than 50 seconds. (0.01 MB PDF) [file pone.0006923.s001.pdf]
